# Synthesis of Intelligent pH Indicative Films from Chitosan/Poly(vinyl alcohol)/Anthocyanin Extracted from Red Cabbage

**DOI:** 10.3390/polym11071088

**Published:** 2019-06-26

**Authors:** Thuy-Vi Vo, Tan-Hiep Dang, Bing-Hung Chen

**Affiliations:** 1 Department of Chemical Engineering, National Cheng Kung University, Tainan 70101, Taiwan; 2 Department of Chemical Technology, Ho Chi Minh City University of Food Industry, Ho Chi Minh City 70000, Vietnam

**Keywords:** poly(vinyl alcohol), chitosan, anthocyanin, pH indicative film, food wrap

## Abstract

In this study, pH indicative films were successfully synthesized from hydrogels made by blending 1% poly(vinyl alcohol) (PVA) and 1% chitosan (CS) with anthocyanin (ATH) and sodium tripolyphosphate (STPP). Particularly, ATH extracted from red cabbage was used as the pH indicator, while STPP was utilized as the cross-linking agent to provide better mechanical properties of the cast films. FT-IR spectra confirmed the existence of the ATH in the cast films. Moreover, the tensile strength, the elongation-at-break, and the swelling indices of the cast films were measured. In general, these properties of pH indicative films were profoundly influenced by the compositions of PVA/CS and the STPP dosage applied in the hydrogels. For example, the tensile strength could change from 43.27 MPa on a film cast from pure PVA hydrogel to 29.89 MPa, if 35% of the PVA hydrogel was substituted with CS. The cast films were applied as a food wrap that could be used to monitor visually the quality of the enwrapped food via the color change of the film upon the variation in pH values of the enwrapped food. In practice, a sequential change in color was successfully observed on the pH indicative films partially enwrapping the pork belly, indicating the spoilage of the meat.

## 1. Introduction

The United Nations adopted 17 Sustainable Development Goals (SDGs) as part of the 2030 Agenda for Sustainable Development on September 25 2015. As an example of one of these goals, SDG 2 is Zero Hunger, which aims to end hunger, to achieve food security, and to promote sustainable agriculture. Indeed, food spoilage and security have always been a predominant concern for human beings since the early days, as the adverse effects of food poisoning could lead to disastrous consequences for human survival. Consequently, relentless efforts have been invested in studying and in the development of proper packaging and preservation of food all around the world [1,2,3,4]. For example, the invention of synthetic polymers has resulted in the mass production of plastic bags and thin-film wraps that are widely applied as food packaging materials. In general, these plastic films have relatively low gas permeability. As such, they often serve as passive barriers to moisture, oxygen and microorganisms to protect the enwrapped food from spoilage. In addition, several SDGs have been proposed to encourage more sustainable consumption and production patterns as well as more sustainable development of our world. Therefore, renewable packaging substitutes, e.g., plastics from renewable biomass sources, are under extensive development to replace the synthetic components in food packaging [1,2,3,4]. Even more, additional components such as antioxidants and colorants are processed into the plastic films [2]. One example is shown in the inclusion of anthocyanins, commonly found as antioxidants and pigments in plants [5], to actively indicate the variation in pH values near the contact between food and package [6].

One aim of this work is to develop a food packaging film made as much as possible from renewable and biodegradable components. Chitosan (CS) is consequently chosen as the substituent to partially replace poly(vinyl alcohol) (PVA) in the packaging film. In addition, anthocyanin (ATH), commonly used as food colorants [5] and extracted from red cabbage in this work, is applied to the cast film for actively monitoring the change in pH values of the enwrapped food.

Chitosan (CS) is a natural polymer derived from chitin and found abundantly in the shells of crabs and shrimps [7,8]. It possesses special characteristics, such as a polycationic nature and chelating properties, as well as a film-forming capability, due to the affluent amino and hydroxyl functional groups in chitosan. Chitosan is found to exhibit many interesting biological activities, such as antimicrobial activity, biodegradability, etc. [7,8,9,10,11]. Adversely, chitosan is not always preferable in polymer processing for film production owing to its inferior mechanical properties, such as lower tensile strength, less elongation-at-break, and Young’s modulus. Hence, chitosan is commonly blended with other polymers with more flexible chains, e.g., poly(vinyl alcohol) (PVA), for further processing of polymer films [8,9,10,12,13,14,15,16].

Poly(vinyl alcohol) (PVA) is a water-soluble biodegradable synthetic polymer with large tensile strength and flexibility, which also allows very low permeability of gases, including O_2_ and CO_2_. PVA can possess a moderate water solubility at an elevated temperature [9,10,16]. With excellent film forming properties, PVA has been widely used in applications as thin films, e.g., for food packaging and packaging for laundry detergent tablets. The composites blended with PVA and chitosan are known to have improved stability, biocompatibility, and mechanical strength, relative to those of pure PVA and pure CS polymers [8,9,10,11,12,13,14,16].

Anthocyanins are natural colorants, water-soluble pigments abundant in plants, such as red cabbage, blueberry, eggplants and flowers. They could be used as a color indicator depending on pH values. In particular, the anthocyanins obtained from red cabbage can vary in color from red to purple and even blue at different pH values, which facilitates the recognition of food quality in terms of pH changes [5,6,8,10,14,15,17,18,19,20,21]. Therefore, the indicative film synthesized from PVA and CS with ATH as the color indicator could express good spectroscopic and physicochemical characteristics and could be applied in intelligent food packaging [14].

In processing thin polymer films, the addition of cross-linking substances, such as sodium tripolyphosphate (STPP), glyoxal, and glutaraldehyde, is necessary to improve the mechanical strength of cast films [22]. These cross-linkers could connect one polymer chain to different macromolecules to eventually form polymer mats. These polymer mats, compared to the less cross-linked ones, exhibit better membrane durability and reduced water absorption [10,14]. Specifically, STPP is capable of forming an ether or ester by cross–linking hydroxyl groups on the same molecules or multiple polymer molecules [14,23].

Solvent casting and hot melt extrusion are currently two of the most popularly applied processing methods for the production of polymer thin-films. The hot melt extrusion process often requires the input of high energy, such as mechanical energy in the form of very high shear stress and/or thermal energy, to ensure the processed polymer in the melt state. As such, this method is not suitable for processing a polymer with heat and/or shear sensitive molecules. In contrast, solvent casting is the predominant method for manufacturing films containing temperature-sensitive ingredients, such as anthocyanin, because the temperature required to evaporate the solvent is often lower than the process temperature of the hot melt extrusion [24].

As aforementioned, PVA/CS composites were chosen as model polymers for the study of pH indicative thin-film wraps in this work. PVA/CS hydrogels have recently been extensively studied for their potential applications, such as in biomedical applications and biomaterials [13,16]. Owing to the polysaccharide nature of chitosan, the percent elongation-at-break decreases significantly with increasing CS content in hydrogels [13]. In this report, the influence of the cross-linking agent, e.g., STPP, and the mixing ratio of PVA/CS on the mechanical properties of the resulted thin films, especially in the changes of tensile strength, the elongation-at-break and water absorption (swelling index), were investigated. The pH indicative films were examined by FT-IR to determine the chemical interaction among polymer components and anthocyanin as well.

## 2. Materials and Methods

### 2.1. Materials

Chitosan (CS, 75%–85% deacetylated), poly(vinyl alcohol) (PVA, *M*w = 89,000–98,000), and sodium tripolyphosphate (STPP) were purchased from Sigma-Aldrich. Hydrochloric acid (37%) and glacial acetic acid were supplied by Merck. All chemicals were of analytical grade and used as received. Deionized water with resistivity greater than 18.2 MΩ-cm was used in sample preparation in this study.

The anthocyanin (ATH) used in this work was extracted from red cabbage (*Brassica oleracea*), a produce harvested from the Lâm Đồng Province of Vietnam, chiefly following the solvent extraction method reported by Fuleki and Francis [25]. Accordingly, the extraction procedure for ATH from red cabbage has been modified in this work and is given as follows. Typically, 150 g of minced red cabbage is macerated and soaked in ca. 303 mL of solvents, composed of absolute ethanol (150 mL) and concentrated HCl (3 mL) in deionized water (150 mL), in an ultrasonic bath for 60 min [10,18,26].

### 2.2. Preparation of pH Indicative Films

The pH indicative films were synthesized with the solvent (solution) casting method using the hydrogels prepared from the mixed solutions of chitosan (CS)/poly(vinyl alcohol) (PVA) incorporated with anthocyanin (ATH) by a procedure revised from that of Stefani et al. [14,19]. STPP was used as the crosslinking agent to the polymer films. All ingredients for making up hydrogels as precursors to the pH indicative films were previously prepared as pure solutions. Notably, the concentrations of polymers and STPP in the parental solutions were 1 wt% for both PVA and CS, and 0.1 wt% for STPP, respectively. Furthermore, the concentration of anthocyanin in the extract was maintained at 86.67 mg/L.

The parental solution of 1 wt% PVA was obtained by dissolving 1 g of PVA in 99 g of deionized water with continuous magnetic stirring at 70 °C. The PVA solution was then allowed to cool down to the ambient temperature, at which it became transparent and quite viscous. Similarly, the 1 wt% CS solution was prepared by dissolving 1 g of chitosan flake in 99 g of 1 wt% acetic acid solution under continuous agitation for 24 h at room temperature.

To study the effect of chitosan and the crosslinking agent STPP on the mechanical properties of the finished pH indicative films, various volume ratios of 1 wt% PVA solution and 1 wt% chitosan solutions were mixed and agitated constantly for 30 min to give the PVA/CS solutions. Subsequently, a proper volume of ATH extract was added into the aforementioned mixed solutions of PVA/CS and stirred for another 30 min at room temperature. The volume of the added ATH solution was fixed at a quarter of the total volume of the final PVA/CS/ATH solutions. For example, 100 mL of PVA/CS/ATH solution was composed of 75 mL PVA/CS solution and 25 mL ATH solution. Afterwards, a suitable volume of 0.1% STPP solution was added to the solutions of PVA/CS/ATH in order to promote the cross-linkage in the resultant hydrogels.

Explicitly, the formulation used for the study on the effect of chitosan dosage on the mechanical properties of pH indicative films was given in proportion as follows: (1) 75 mL PVA/CS solution, in which various volume ratios of PVA solution to CS solution were used; (2) 25 mL ATH extract solution; and (3) 6 mL of 0.1% STPP solutions. Similarly, the formulation for the study on the effect of the crosslinking agent STPP on the mechanical properties of pH indicative films was given in proportion as follows: (1) 75 mL PVA/CS solution, i.e., the volume ratio of PVA solution to CS solution kept at 75/25; (2) 25 mL ATH extract solution; and (3) the predetermined amount of 0.1% STPP solutions.

The pH value of the hydrogel, consisting of PVA/CS/ATH/STPP, was adjusted to 6 with 1.0 M NaOH. Finally, the pH indicative film was attained by casting the obtained hydrogel (50 mL) in Petri dishes (120 mm diameter), followed by the removal of solvent from the cast film by placing these Petri dishes in an oven at 45 °C for 72 h. All the experiments reported in this work were performed at least in triplicate.

### 2.3. Characterization of pH Indicative Films

A universal tensile testing instrument, Model RTC-1210A, from A&D Company (Tokyo, Japan) was employed to measure the tensile strength (TS) and the percent elongation-at-break of the pH indicative film using the method described in ASTM D882 [27]. The specimens for the universal tensile testing were prepared in rectangular strips with a dimension of 100 mm (length) × 10 mm (width) × 50 μm (thickness). The speed of testing was chosen at 50 mm/min.

The swelling index was determined by the gravimetric method. The prepared films were cut into coupons of 30 × 30 mm^2^. These strips of pH indicative films were weighed individually and subsequently immersed in 100 mL of deionized water for various periods of time at room temperature. At each time interval (0.5, 1, 2, 5, 10, 15 and 20 min), sample specimens were taken out, dried and weighed. Consequently, the swelling index (*SI*) was calculated using Equation (1):

(1) $$SI\;(\%)=\frac{m_{1}-m_{0}}{m_{0}}\times 100$$

where *SI* is the welling index, namely the percentage of water adsorption by the film, as well as *m_0_* and *m_1_* are the weights (g) of the film initially and at the given time *t*, respectively.

The functional groups of *as*–prepared indicative films were characterized by using the FT-IR Spectrometer (Bruker Tensor 27, Germany) in the Institute of Chemical Technology (Ho Chi Minh City, Vietnam). The FT-IR spectra of the samples diluted in KBr pellets were recorded in the 4000–400 cm^−1^ range with a resolution of 4 cm^−1^. In addition, the morphology of the films was observed using a Field Emission Scanning Electron Microscope (FE–SEM, Hitachi S–4800, Tokyo, Japan) at the Research Laboratories of Saigon High-Tech Park (SHTPLABS, Ho Chi Minh City, Vietnam)

### 2.4. Coloration of pH Indicative Films

The coloration of the pH indicative films was demonstrated in the buffer solutions with pH values from 1 to 13. The films were cut into small coupons of 2 × 2 cm^2^ and submerged in buffer solutions. Usually, the color change could be observed and recorded within 30 s.

## 3. Results

### 3.1. Synthesis and Chemical Characteristics of pH Indicative Films

**Figure 1:**
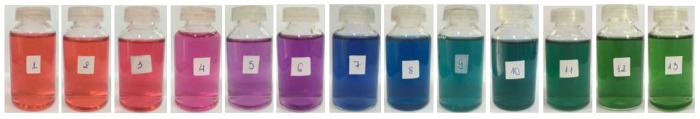
Color change of anthocyanin extract from pH 1 (**left**) to pH 13 (**right**).

Anthocyanin (ATH) was successfully extracted from red cabbage. Figure 1 showed clearly the color change in buffer solutions with an ATH concentration at 86.67 mg/L from pH 1 to pH 13. One advantage associated with the use of anthocyanin as a pH indicator is reflected from their obvious change in color upon contact with different pH values. For example, their colors are red or pink in the acidic solutions, and blue or green in alkaline solutions. Consequently, the ATH was used as a pH indicator and formulated to cast the pH indicative films composed of PVA, CS and STPP.

**Figure 2:**
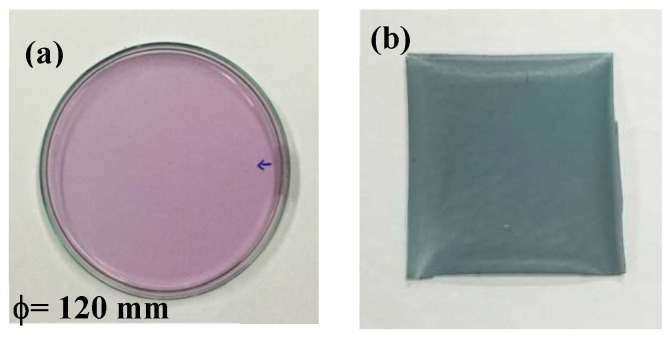
Poly(vinyl alcohol) (PVA)/chitosan (CS)/anthocyanin (ATH) hydrogel and the cast film: (**a**) before and (**b**) after the evaporation of water.

Prior to the addition of the cross-linker STPP, the solution consisting of PVA/CS/ATH in a volume ratio of 26.25/48.75/25, viz. (75 × 0.35)/(75 × 0.65)/25, at pH 6.1 exhibited a pink-magenta color (Figure 2a), similar to that of the ATH extract at pH 6 (Figure 1). The hydrogel was gradually formed in the aforementioned PVA/CS/ATH solution after the slow addition of STPP in a dose at the volume ratio of STPP solution to PVA/CS/ATH solution at 6%. The pH indicative film was cast after water had been removed from the hydrogel with an oven-drying process at 45 °C for 72 h. As aforementioned, the film was cast from 50 mL of hydrogels in a Petri dish of 12 mL in diameter. Explicitly, this dry film was composed approximately of 123.8 mg PVA, 230 mg CS, 1.02 mg ATH and 2.83 mg STPP.

Interestingly, the color of the resultant film changed from pink-magenta as a wet hydrogel (Figure 2a), to sea-green as a dried cast film (Figure 2b). It is surmised that color changes during the oven-drying process are mainly the result of the pH change in the hydrogel owing to the gradual evaporation of water from the PVA/CS/ATH/STPP hydrogel, in which both sodium hydroxide and acetic acid existed simultaneously in the gel.

**Figure 3:**
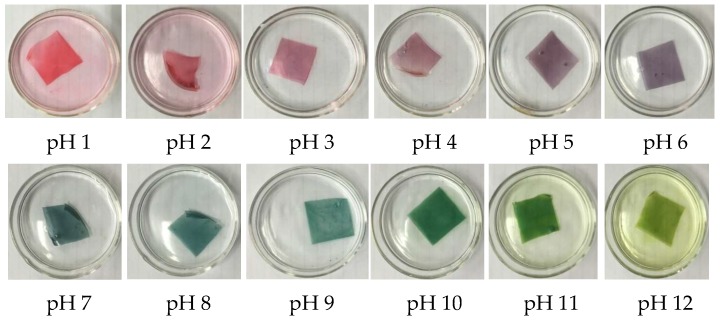
Coloration of the cast pH indicative films submerged in solutions at different pH values.

The coloration of the cast pH indicative films submerged in buffer solutions with pH values from 1 to 12 is shown in Figure 3. These films were sensitive to the pH values of the solutions. Commonly, the color change on these pH indicative films occurred very quickly, in usually less than 30s. The coloration of these films could be attributable to the transformation in the chemical structure of ATH molecules with respect to the pH values of the solution [5,14,26]. At pH 1, this film shows a reddish color, and markedly changes its color from reddish to purple as the pH is increased to 6. It is blue at pH 7 and 8, but changes to sea green at pH 9. With an increase of pH to 12, this film becomes yellow-green fast. The vivid color changes of the prepared films against the pH values have enabled their applications as pH indicators in food packaging, and this will be demonstrated in the latter part of this report.

**Figure 4:**
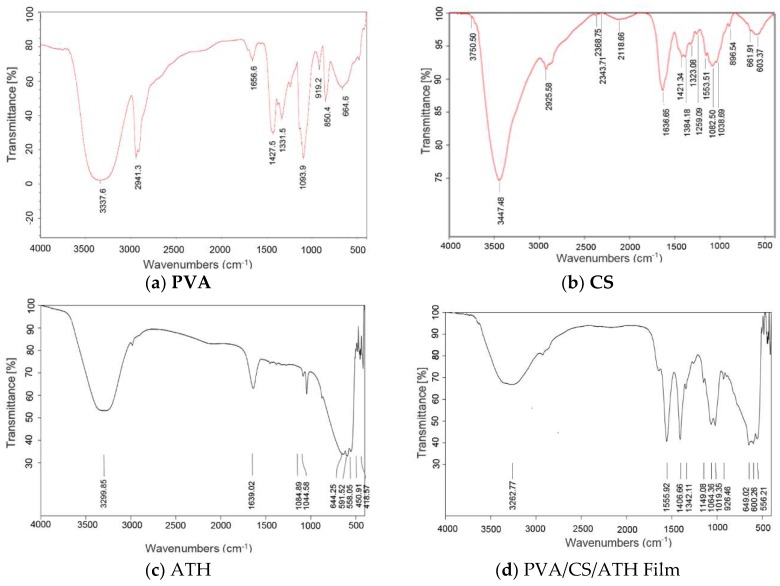
FT-IR spectra of (**a**) PVA, (**b**) chitosan, (**c**) anthocyanin, (**d**) PVA/CS/ATH film.

The FT-IR spectra of PVA, CS, ATH and the PVA/CS/ATH film are shown in Figure 4. The film examined was synthesized from the ATH-containing hydrogel with a volume ratio of PVA/CS=35/65. On the FT-IR spectrum of PVA, peaks corresponding to 3337.6 cm^−1^ and 1427.5 cm^−1^ are attributed to the stretching and the bending vibrations of –OH group, while the bands at 1331.5 cm^−1^ and 2941.3 cm^−1^ are the result of –CH_2_ stretching vibrations. The peak appearing at 1656.6 cm^−1^ arises from the crystallized water molecules in the polymer. The bands from 919.2 to 1093.9 cm^−1^ feature the oscillations of the C–O–H group [12,28]. For pure chitosan (CS), the absorption at 3447.48 cm^−1^ corresponds to the stretching –OH group, and the band at 2925.58 cm^−1^ is one characteristic of the –CH_2_ group bonded to –OH. The peak at 1636.65 cm^−1^ is attributed to C–O stretching of the acetyl group (amide I). Furthermore, the peak at 1421.34 cm^−1^ is the bending vibration of the –C–OH group, while that of 1384.18 cm^−1^ is the result of the knife in the plane of the –OH group. The regions from 1153.51 to 1038.69 cm^−1^ are the characteristics of the symmetric oscillation of the C–O–C group [14,28]. As for the FT-IR spectrum of anthocyanin, the uptake at 3299.85 cm^−1^ relates to the –OH group, and the peak at 1639.02 cm^−1^ represents the C=C aromatic ring, bands from 1044.58 to 1084.89 cm^−1^ arise from the oscillations of the C–O–C group [14].

The FT-IR spectra of the PVA/CS/ATH pH indicative film, shown in Figure 4d, obviously contain some characteristics of PVA, chitosan and anthocyanin. The broadened peak at 3262.77 cm^−1^ is a result of the stretching of the –OH group. The band at 1555.92 cm^−1^ is attributable to the bending of the -NH bond of amine, which can be explained by the fact that the amine group –NH_2_ in chitosan has reacted to form the amine -NH (II). The bands from 1149.08 to 1406.66 cm^−1^ stand for the deformation of the –CH_2_ group in –CH_2_OH. In addition, the adsorption bands from 926.46 to 1064.36 cm^−1^ are typical of the oscillation of the C–O–C group.

**Figure 5:**
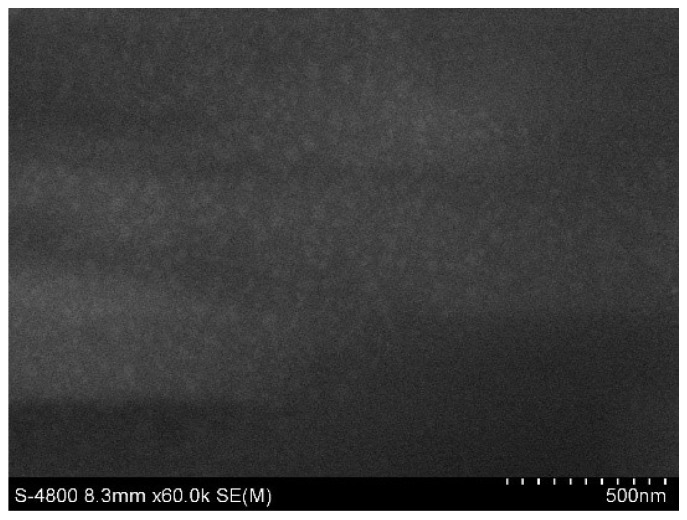
SEM micrograph of PVA/CS/ATH film synthesized from the ATH-containing hydrogel with a volume ratio of PVA/CS = 35/65.

The surface morphology of the polymer film was probed by SEM (Figure 5). The surface of the PVA/CS/ATH film was quite smooth. No crystal grains could be detected at a magnification of 60,000 times, suggesting that the membrane components were uniformly blended.

### 3.2. Effect of STPP Crosslinking Agent on Physical Properties of pH Indicative Film

**Figure 6:**
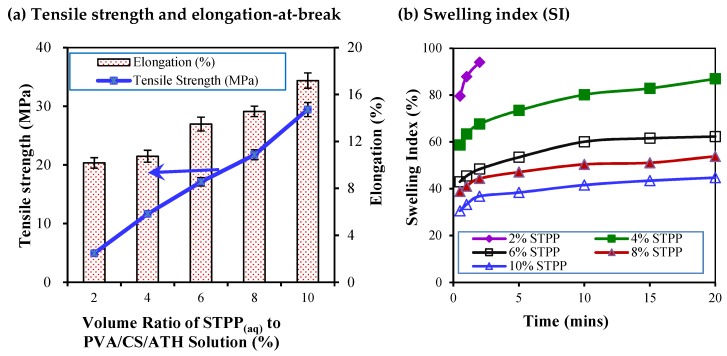
Effect of sodium tripolyphosphate (STPP) dosage on the tensile strength and the percent elongation-at-break of the cast films (**a**) and the swelling index (SI) of the cast films (**b**).

PVA/CS hydrogels have recently been extensively studied for their potential applications, e.g., in biomedical applications and biomaterials [13,16]. Chitosan is a kind of natural polymer, derived from sustainable resources [8]. Owing to the polysaccharide nature of chitosan, the percent elongation-at-break decreases significantly with an increasing CS content in hydrogels [13]. Therefore, addition of biodegradable and flexible PVA to CS becomes an alternative for the cast films. Stefani et al. [14] studied the PVA/CS/ATH film as a time-temperature indicator. In their work, the composition of CS and PVA in the cast film was not varied but, instead, maintained constant at 70/30 along with a constant dose of STPP [14]. It is known that the crosslinker could influence the structural strength of the hydrogel and the resultant films. Hence, the effect of STPP dosage on the mechanical properties of the resultant PVA/CS/ATH films was studied in this work (Figure 6a). Similarly, Figure 6b shows the effect of STPP dosage on the time-dependent swelling of the cast films. Notably, the results shown in Figure 6 were obtained from experiments with PVA/CS at 25/75. In general, the tensile strength and the percent elongation-at-break increased with an increasing amount of STPP added to the hydrogels. Similarly, the swelling ratio of the cast films was significantly suppressed with more STPP initially present in the hydrogel.

The addition of the cross-linking agent STPP to the hydrogels is required in casting PVA/CS/ATH films. In practice, films cast without STPP added to the hydrogels were easily smashed into species when they were peeled out from petri dishes after solvent vaporization. This could be probably attributable to very weak cross-linking bonds formed in the cast films. Thus, cast films without STPP added could not be examined with the tensile testing instrument.

With the amount of STPP added increasing from 2% to 10%, the tensile strength is enhanced from 4.9 MPa to 29.4 MPa, i.e., a sextuple increment in tensile strength, and the elongation-at-break is increased from 10.2% to 17.2%. STPP with a chemical formula of Na_5_P_3_O_10_ is a penta-anion, and can possibly create electrostatic bonds with some polycations, such as chitosan [23]. As such, STPP could enhance the molecular entanglement among PVA and chitosan entrapped with ATH molecules, thus leading to a more-difficult-to-break film that possesses increasing tensile strength. On the other hand, with more STPP they tend to form more tightly integrated blocks with larger structures of polymer complexes resulting in a more plastic-like nature of the synthesized films and an increased elongation-at-break of the cast films. In brief, the addition of the crosslinking agent will increase the entanglement of chitosan and PVA, namely leading to larger tensile strength and more elongation-at-break [14].

The swelling index (SI) could be used as an indicator for the water absorptivity of the pH indicative film. In general, a greater extent of cross-linkage in pH indicative films would improve the mechanical properties and the water resistance of the films. Therefore, it is expected that the cast films would absorb less water with more STPP initially introduced into the CS/PVS/ATH hydrogels. For example, the resultant film with 2% STPP was shown to have the largest water absorptivity as its SI increased to 94% in 2 min. Afterwards, the film was broken down into small pieces. Therefore, it is impossible to measure the weight of the water-adsorbed films submersed in water for more than 3 min. Increasing the dosage of STPP to 10%, the water absorptivity of the film could decrease significantly, while its SI was increased from 30% to 43.5% after being immersed in water from 0.5 min to 20 min. After a 20-min duration of submersion in water, this pH indicative film remained almost in shape. In brief, with addition of 4%–10% STPP, the resultant pH indicative films remained intact after a prolonged duration of submersion, e.g., 20 min. Notably, the synthetic films became less translucent with the initial STPP dosage more than 6%, possibly owing to the increasing coherent linkage of molecules. Consequently, STPP dosages of 2% and 6% were chosen for further study on the effect of the volume ratios of PVA/CS in the hydrogel on the mechanical properties of synthesized pH indicative films.

In general, as the dosage of the crosslinking agent STPP used in PVA/CS/ATH films increased, the SI values of these films decreased. Alternatively speaking, as the cross-linking grids inside these cast films get stronger with an increasing STPP dosage, the process of water molecules imbibed into cast films could become more difficult. Fewer ATH molecules would be leached out from the cast films. Likewise, when the duration of the submersion of cast films in water increased, the absorption amount of water and the ATH quantity leached out from cast films increased accordingly.

### 3.3. Effect of the PVA/CS Ratio on Mechanical Properties of the pH Indicative Film

**Figure 7:**
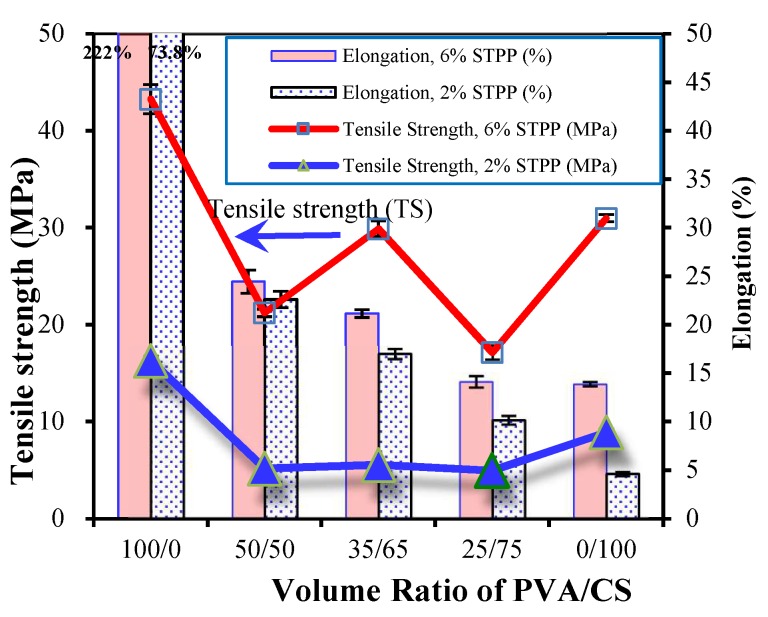
Effect of mixed volume ratios of PVA/CS on the tensile strength and percent elongation-at-break of the synthesized pH indicative films.

As aforementioned, the aim of this work is to explore how to use more sustainable components, e.g, chitosan (CS), in packaging films. Accordingly, the effect of the CS dosage in the PVA/CS mixed solutions on the resultant pH indicative films was studied. The mechanical properties of the pH indicative films with 2% and 6% STPP, including the ultimate tensile strength (TS), the elongation-at-break and the swelling index, are summarized in Figure 7.

It would seem that films cast from PVA only possessed a larger elongation-at-break, namely 222% and 73.8% with initial STPP dosages at 6% and 2%, respectively, in contrast to 13.9% and 4.62% for films cast from chitosan only. Chitosan is a linear polysaccharide and, specifically, a random copolymer of d-glucosamine and N-acetyl-d-glucosamine, while PVA is often an atactic polymer. From a molecular point of view, the rigid 6-membered ring structures of d-glucosamine and N-acetyl-d-glucosamine in the main chain make chitosan more difficult to rotate and less flexible than linear PVA [16,29]. Hence, the elongation of pure CS mat is expected to be less than that of the PVA mat. Likewise, the tensile strength of pure PVA films is larger than that of pure CS [9,10]. In general, the differences between CS films and PVA films in the elongation-at-break and the tensile strength become more obvious with more STPP initially introduced to the PVA/CS hydrogels.

Interestingly, the trends in elongation and tensile strength of pH indicative films are not so clear with the CS doses in PVA/CS hydrogels (Figure 7). Generally, the elongation-at-break decreases with an increasing CS amount in the PVA/CS films. In contrast, the tensile strength of PVA/CS films was lower than those of pure PVA films. With 2% STPP, the tensile strength of PVA/CS films cast from mixed hydrogels with PVA/CS mixed ratios at 50/50, 35/65 and 25/75 were found at 5.18 MPa, 5.54 MPa and 4.94 MPa, respectively. That is, the film cast from PVA/CS at 35/65 possesses the relatively highest tensile strength. This becomes more obvious in the tensile strength of PVA/CS films with 6% STPP. At a PVA/CS mixed ratio at 35/65, the tensile strength was measured as 29.9 MPa, compared to 21.2 MPa and 17.1 MPa for those films with a PVA/CS ratio at 50/50 and 25/75.

**Figure 8:**
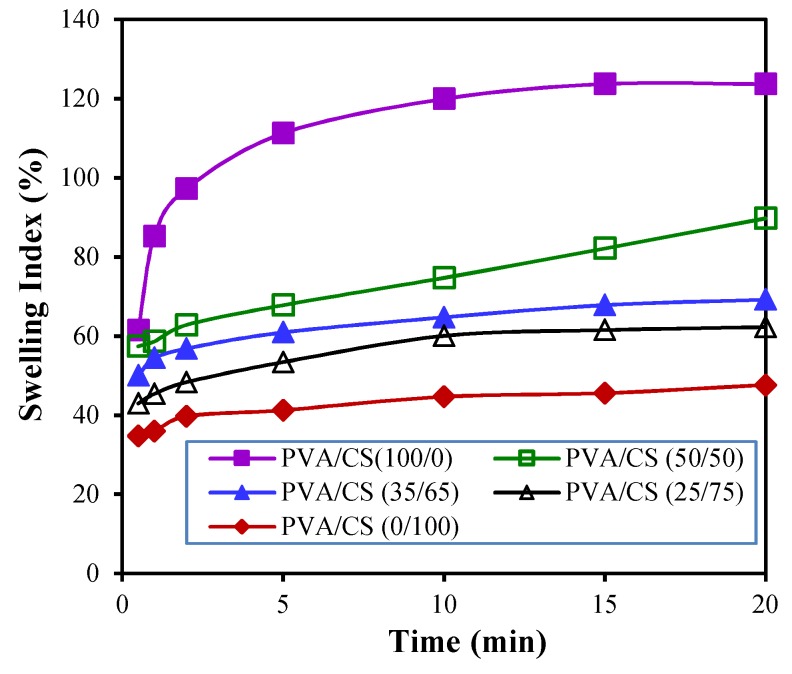
Effect of volume ratios of PVA/CS on the swelling index of the synthesized pH indicative films dosed with 6% STPP.

As shown in Figure 6b, films cast with 2% STPP swelled quickly. Hence, only films cast with 6% STPP proceeded to the swelling test. The absorption amount of water by pH indicative films varied and was dependent on the mixed volume ratios of PVA/CS in the films. In general, the adsorption kinetics of water by these films grows exponentially with more submersion time. Moreover, an increasing CS content in indicative film results in a smaller swelling index at the same submersion time (Figure 8). For example, more water was imbibed into the PVA film than others, as reflected in the relatively larger SI values of PVA films ranging from 61.5% to 123.7%. This can be attributed to the hydrophilic nature of PVA polymer. The lowest SI values were found around 34.8% to 47.7% on the films containing chitosan only. The SI values of PVA/CS indicative films are generally larger when more PVA is present in the mixed hydrogels of PVA/CS. For example, the SI values of PVA/CS film with a mixed PVA/CS ratio of 25/75 for hydrogel increase from 43% at 0.5 min to 62.3% at 20 min, while the SI values of films cast from the hydrogel (PVA/CS = 35/65) increase from 50.1% at 0.5 min to 69.2% at 20 min. That is, the water absorptivity of PVA/CS films increases with increasing PVA content in hydrogels. Generally, the as-synthesized PVA/CS films could absorb water quickly and possessed good tensile strength. Taking the mechanical properties into account, the film cast from the mixed hydrogel of PVA/CS = 35/65 is deemed as suitable for a pH indicative food-packaging application since the food quality could be visually observed by the color change of the PVA/CS films.

### 3.4. Application of PVA/CS/ATH pH Indicative Film for Food Packaging

**Figure 9:**
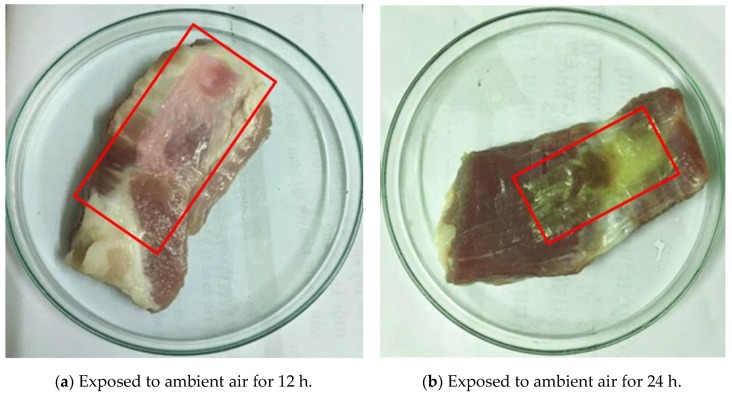
Color change of PVA/CS/ATH films in contact with raw pork belly slices exposed to ambient air for 12 h and 24 h.

The cast PVA/CS/ATH pH indicative films were applied to partially wrap pork belly slices, which were covered with Petri dishes and exposed to ambient air for 12 h and 24h. The covered portions by films are highlighted and enclosed in the red rectangles on Figure 9, whereas the rest were exposed to air. The partially wrapped meat was intentionally spoiled in the ambient conditions to observe the successive color change of the pH indicative films during the spoilage of the meat.

The cast film was initially translucently sea-green, as shown in Figure 2. After being exposed to ambient air for 12 h, the film wrapping the meat becomes pink, indicating an acidic condition near pH 5–6 on the surface of pork slices. The mucilaginous surfaces of the meat slices appeared as with dense layers of bacteria and fungi. The funky and sour flesh odor could be smelled, as the meat was contaminated with microorganisms that could decompose proteins and fat into polypeptides, amino acids, fatty acids and other compounds with characteristic odors, such as hydrogen sulfide, indole, skatole, butyric acid, etc. With further exposure in the ambient conditions for another 8 h, the pork meat turned dark-brown and softer, while the pH indicative film became yellowish with pale green, showing it in the slight alkaline range. In brief, the pH indicative film applied to wrap the meat successfully showed the sequential change in color during the meat spoilage.

## 4. Conclusions

The pH indicative films of PVA/CS/ATH have been successfully cast from hydrogels by blending 1% PVA solution and 1% CS solution in various volume ratios, doped with ATH as an indicator, and mixed with STPP as a cross-linker. The tensile strength, the elongation-at-break, and the swelling indices of the cast films have been measured. These films possess the satisfactory mechanical properties and exhibit a rapid color change in pH buffers. For example, the film synthesized from the mixed hydrogels made of 1 wt% PVA solution of 35 mL, 1 wt% CS solution of 65 mL and 0.1 wt% STPP of 6 mL, was found to have the tensile strength at 29.89 MPa and a percent elongation-at-break near 21.13%. FT-IR spectra of the cast films confirmed the existence of the ATH in the cast film. The PVA/CS/ATH pH indicative films were applied to partially wrap slices of pork belly, which were exposed to ambient air for 12 h and 24 h. These films have successfully shown a series of color changes with regard to exposure time, corresponding to the observation on food spoilage.
